# Effects of tadalafil on sexual behavior of male rats induced by chronic unpredictable mild stress

**DOI:** 10.1093/sexmed/qfad019

**Published:** 2023-05-26

**Authors:** Heng Wang, Xue Liu, Ziheng Zhang, Ziyang Han, Yongsheng Jiang, Yu Qiao, Tao Liu, Jianhuai Chen, Yun Chen

**Affiliations:** Department of Andrology, Jiangsu Province Hospital of Chinese Medicine, Affiliated Hospital of Nanjing University of Chinese Medicine, Nanjing 210029, China; Department of Andrology, Jiangsu Province Hospital of Chinese Medicine, Affiliated Hospital of Nanjing University of Chinese Medicine, Nanjing 210029, China; Department of Andrology, Jiangsu Province Hospital of Chinese Medicine, Affiliated Hospital of Nanjing University of Chinese Medicine, Nanjing 210029, China; Department of Andrology, Jiangsu Province Hospital of Chinese Medicine, Affiliated Hospital of Nanjing University of Chinese Medicine, Nanjing 210029, China; Department of Andrology, Jiangsu Province Hospital of Chinese Medicine, Affiliated Hospital of Nanjing University of Chinese Medicine, Nanjing 210029, China; Department of Andrology, Jiangsu Province Hospital of Chinese Medicine, Affiliated Hospital of Nanjing University of Chinese Medicine, Nanjing 210029, China; Department of Reproductive Center, Affiliated Huai'an No. 1 People's Hospital of Nanjing Medical University, Huai'an 223001, China; Department of Andrology, Jiangsu Province Hospital of Chinese Medicine, Affiliated Hospital of Nanjing University of Chinese Medicine, Nanjing 210029, China; Department of Andrology, Jiangsu Province Hospital of Chinese Medicine, Affiliated Hospital of Nanjing University of Chinese Medicine, Nanjing 210029, China; Department of Andrology, Jiangsu Province Hospital of Chinese Medicine, Affiliated Hospital of Nanjing University of Chinese Medicine, Nanjing 210029, China

**Keywords:** chronic unpredictable mild stress, psychogenic erectile dysfunction, sexual behavior, rat model, tadalafil

## Abstract

**Background:**

Few studies have investigated psychogenic sexual dysfunction including psychogenic erectile dysfunction (pED); the effect of tadalafil on sexual behavior of male rats induced by chronic unpredictable mild stress (CUMS) remains unclear.

**Aim:**

The aim was to explore the influence of CUMS on sexual behavior of male rats and the effects of tadalafil on that.

**Methods:**

Adult male rats were divided into 3 groups, including the normal group without CUMS, the model group with 6 weeks’ CUMS, and the tadalafil group with treatment of tadalafil during CUMS. CUMS consists of water deprivation, food deprivation, stroboscopic lightning, white noise, cage tilting, weeding packing, and housing 2 unfamiliar rats. The apomorphine test and vaginal smear test were conducted with the aim to screen out male rats with good erectile function and make preparation for the sexual behavior test, respectively.

**Outcomes:**

At the end of the study period, the level of anhedonia and sexual function were evaluated by the sucrose preference test, sexual behavior test, and measurement of serum testosterone, dopamine, and 5-HT.

**Results:**

Sucrose preference showed significant decrease in rats after CUMS. The intromission ratio and total intromission frequency decreased significantly, while the mount latency and ejaculation latency prolonged significantly in CUMS-induced rats when compared with normal rats. Meanwhile, the treatment of tadalafil reversed the level of anhedonia and sexual function in CUMS-induced rats. However, there were no statistical differences in the levels of serum testosterone, dopamine, and 5-HT among groups.

**Clinical Implications:**

The study constructed an animal model that can provide clinical insights into the mechanism of psychogenic sexual dysfunction and supports the application of tadalafil in pED therapy.

**Strengths and Limitations:**

We found that CUMS-induced rats exhibited anhedonia and poor sexual function that could be prevented by tadalafil administration. Future research needs to construct the standard of pED model and explore the mechanism of tadalafil on central nervous system.

**Conclusion:**

Tadalafil could prevent the changes of depression and poor sexual function in rats induced by CUMS, and the method of CUMS and the sexual behavior test should be used in the future for pED modeling.

## Introduction

Erectile dysfunction (ED) is a common male sexual dysfunction, which is defined as the constant inability to attain or maintain penile erection sufficient for successful sexual performance.^1^ Psychogenic ED (pED), the most common type of ED, is usually caused by psychological and social factors (eg, depression, anxiety, disharmonious couple relationship) without organic lesion of the penis.^2^ Psychological factors play an important role in the occurrence and development of ED, especially pED.^1^ To date, various types of rodent models, especially ED rats and mice, have been developed and applied in the studies about the cellular and molecular mechanisms of ED.^3^ Aged rats are usually employed as an age-induced ED model to explore the relationship between aging and erectile function. In the measurement of intracavernosal pressure (ICP), compared with young normal rats (3 months of age), aged rats (18 months of age) demonstrated a decreased ICP and ICP-to-systemic-mean-arterial-pressure ratio, which reflected poor erectile function.^4^ Streptozotocin-induced diabetic rats are applied into the research of ED caused by endothelial dysfunction, diabetic neuropathy, or blood vessel injury. An impaired NO-cGMP pathway or oxidative stress damage may be responsible for the penile erection of diabetic-induced ED rats.^5^ In addition, a variety of animal models including cavernous nerve injury–induced ED and hypercholesterolemia/hyperlipidemia have been widely applied in understanding pathophysiology of ED and usage of sexual medicine.^6^

The previously mentioned models are mainly organic and can be diagnosed by an apomorphine test (observing the rat’s penile erection through injecting apomorphine subcutaneously in the back of the neck) or ICP test.^7^^,^^8^ The establishment of a pED model that is known as nonorganic is more complex, and a more stable and effective pED model has been attracting researchers’ interests.^6^ Although the mechanism of pED still remains unclear, some methods for constructing pED models have been reported. In fact, the pED model is usually based on the depression model through different stress methods, which can drive the rodents into depression. The methods of immobilization,^9^ electric foot shocks,^10^ and immersion in cold water^11^ have been reported in previous studies. The stressed rats were found to have lower intromission in sexual activity compared with the normal rats. However, the single stress used in previous studies is not stable, and some methods may result in injury to the rats’ bodies.^12^ Therefore, chronic unpredictable mild stress (CUMS) has been adopted by more researchers, and some central mechanisms have been explained based on this protocol. Altered brain activities were identified in the medial preoptic area, amygdala, and salience network, and dopamine D2 receptors were found to participate in the central regulation of penile erection in pED rats.^13^^,^^14^ Male rat sexual behavior has been extensively studied. It can evaluate not only erectile function, but also sexual motivation, the neurobiology of ejaculation, and the refractory period after ejaculation. Therefore, it has a high relevance of human sexuality^15^ and has contributed to a better understanding and treatment of sexual function.^16^

Tadalafil (Eli Lilly) has prominent effect on various types of ED. However, few studies have reported the role of tadalafil in pED. In this study, we explored the effect of tadalafil on CUMS-induced rats. The sucrose preference test, sexual behavior test, and apomorphine test were adopted to assess the depression state and sexual function in rats. Finally, the levels of serum concentrations of testosterone, dopamine, and 5-HT, which have relationship with depression and sexual function, were measured.

## Methods

### Establishment and treatment of rats

Male Wistar rats (weighing 180-220 g, 8-10 weeks old) and female Wistar rats (weighing 180-200 g, 8-10 weeks old) were purchased from the Beijing Weitong Lihua Experimental Animal Technology Co, Ltd. Animal experimental operations were approved by the Animal Care Committee of the Experimental Animal Center of Nanjing University of Chinese Medicine. The rats were raised in a standard barrier facility in accordance with the National Institutes of Health Guide for the Care and Use of Laboratory Animals.

**Figure 1:**
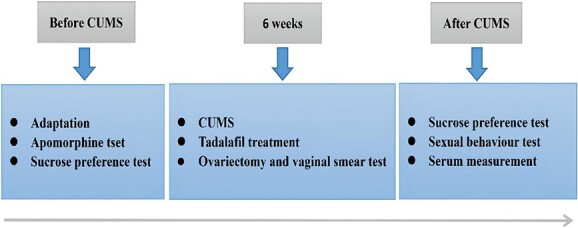
Timeline and schedule of experiments.

During the first week of adaptation, male rats were selected when they had normal erectile function (engorged penis fully exposed in apomorphine test) and sucrose preference up 60%. Then they were randomly divided into 3 groups as the normal group, the model group, and the tadalafil group (n = 8 per group). In the end, in the normal group remained 6 (2 had no intromission during the sexual behavior test were excluded), in the model group remained 7 (1 couldn’t tolerant stress and died during CUMS), and in the tadalafil group remained 7 (1 died owing to gavage mistake). Besides the normal group (4 rats per cage), rats in other 2 groups were single housed in cages (30 × 20 × 20 cm) and exposed to the CUMS for the following 6 weeks. Meanwhile, rats in the tadalafil group were administered tadalafil by oral gavage once daily at a dose of 5 mg/kg before stresses per day. Tadalafil was dissolved in normal saline (0.5 mg/mL) (Figure 1). In the CUMS protocol, we mainly used the stresses of changing rats’ living environment. In the daytime, we adopted water deprivation (24 hours), food deprivation (24 hours), stroboscopic lightning (8-12 hours, 5 Hz), and white noise (4-6 hours, 90 dB), while during the night, we employed cage tilting (tilting the cage into a 45° position for 12 hours), weeding packing (wetting the bedding by pouring water into the cage for 12 hours), and housing 2 unfamiliar rats (12 hours). To avoid the tolerance of rats, each stress would not repeat in 3 continuous days, and in each week, there were 2 half days without stress.^12^^,^^14^^,^^17^

### Ovariectomy and induction of estrus in female rats

We conducted ovariectomy in female Wistar rats after they reached sexual maturity. Then vaginal smear test was performed for 5 days to confirm that they were not in estrus for the following artificial induction of estrus. More than 7 days after surgery, 20 μg estradiol benzoate and 500 μg progesterone (Huamu Animal Health Product Co, Ltd) (both dissolved in 0.1 mL sesame oil) were subcutaneously injected to induce estrus in female rats 48 hours and 4 hours before the sexual behavior test, respectively.^13^

### Vaginal smear test

After the ovariectomy surgery or induction of estrus, the vaginal smear test was conducted to confirm that they were in or not in estrus. Procedures were as follows: (1) a moist cotton swab with a diameter of 0.5 to 1.0 cm was inserted into a female rat’s vagina and gently rotated several times; (2) the cotton swab was removed from vagina and applied lightly on a glass slide and waiting to air dry at room temperature; (3) the glass slide was put in toluidine blue staining solution (ServiceBio) for 3-10 minutes after it was completely dry; (4) the stained slides were taken out and excess dye were washed off with clean water until they were dry; and (5) the smears were viewed under the microscope and judgments were made according to the typical characteristic of each estrous cycle^18^ (Supplementary Figure 1C-E).

### Sucrose preference test

In the study, the sucrose preference test was performed from 8 pm to 9 pm. It is necessary to ensure that the test is performed at the same time before and after CUMS. The test was divided into 3 days. On the first day, each male rat was administered 2 bottles of 1% (w/v) sugar water (Sucrose-BioReagent; Beyotime). One bottle of 1% sugar water and 1 bottle of pure water were adopted on the second day, and the positions were switched after 12 hours to avoid position interference. On the last day, the food and water were deprived in the daytime for 12 hours, and then 2 preweighed bottles were administered, one with 1% sugar water and the other with pure water (change position within 30 minutes). The weights of 2 bottles were recorded again after 1 hour, and the preference rate of sugar water was calculated: sugar preference rate = sugar water intake / (sugar water intake + pure water intake).^19^

### Apomorphine test

Before CUMS, the apomorphine test was carried out to screen out male rats with good erectile function. First, male rats were individually placed in clear observation boxes (30 × 30 × 30 cm) for 10 minutes of adaptation. Then the rats were injected subcutaneously in the back of the neck with apomorphine (Sigma-Aldrich) (80 μg/kg). Each rat was observed by 2 researchers (Z.Z. and Y.J.)who were blind to the specific group at the same time for 30 minutes. A successful erection was described as an engorged glans penis and distal shaft.^7^ After each round, boxes were cleaned and 75% of alcohol was sprayed to eliminate odor interference.

### Sexual behavior test

Considering the habits of Wistar rats, the sexual behavior test was performed in the night. It was crucial to make sure that the environment was quiet and the light was made dark yellow. Before 10 minutes of the test beginning, each male rat was placed in a cage (30 × 40 × 20 cm) for adaptation. Then a female rat in the estrus cycle was placed in the cage, and the following 30 minutes of sexual activity was recorded on video (Supplementary Figure 1A, B). The following parameters were recorded^20^: mount latency (ML) (latency to the first rode straddles); ejaculation latency (EL) (the time from the first insertion of a male mouse to ejaculation); total mount frequency (TMF) (total number of mounts in 30 minutes); total intromission frequency (TIF) (total number of intromissions in 30 minutes); and intromission ratio (IR), that is, IR = TIF/(TMF + TIF). Specially, if ejaculation did not happen in the test, the EL was equal to 1800 seconds.^21^

### Serum testosterone, dopamine, and 5-HT measurement

After rats were anesthetized with isoflurane successfully, blood samples were taken from the abdominal aorta and centrifuged to obtain serum (4°C, 2000 *g*, 20 minutes). The serum was subpackaged into centrifuge tubes and kept at −80°C for use. The serum concentrations of testosterone, dopamine, and 5-HT were measured using enzyme-linked immunosorbent assay kits (Mlbio) according to the manufacturer’s instructions. Samples were detected by a microplate reader (Biotek).

### Statistical analysis

Data were analyzed with GraphPad Prism version 8.0 (GraphPad Software) and presented as mean ± SEM. The Kolmogorov-Smirnov test was used to determine the normal distribution of variables. One-way analysis of variance followed by the least significant difference post hoc test was used to analyze the significant differences. *P <* .05 was considered statistically significant.

## Results

### CUMS induced anhedonia and reduced sexual function in male rats, which could be prevented by tadalafil

**Figure 2:**
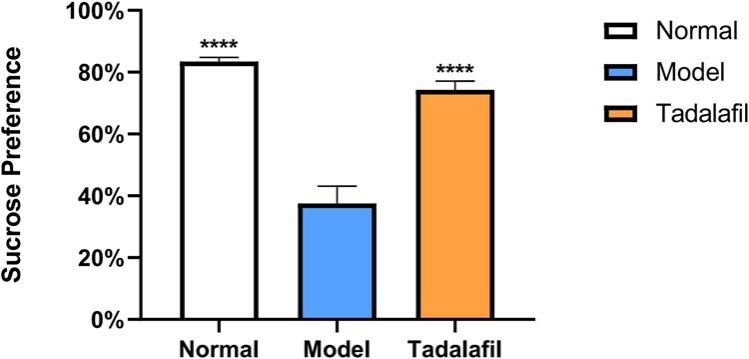
Sucrose preference test. Chronic unpredictable mild stress (CUMS) after 6 weeks obviously led to drop of sucrose preference. Tadalafil treatment reversed the CUMS-induced decrease in sucrose preference. ^*^^*^^*^^*^P < .0001 compared with the model group. Values are mean ± SEM.

One-way analysis of variance revealed significant differences in 3 groups in terms of sucrose preference (*F*_2,17_ = 39.410, *P <* .0001) and ML (*F*_2,17_ = 3.629, *P <* .05), TIF (*F*_2,17_ = 17.010, *P <* .0001), TMF (*F*_2,17_ = 1.670, *P =* .218), IR (*F*_2,17_ = 64.410, *P <* .0001), EL (*F*_2,17_ = 3.911, *P <* .05), and EF (*F*_2,17_ = 3.906, *P <* .05). Decreased sucrose preference was identified in the model group after 6 weeks’ CUMS when compared with the normal group (*P <* .0001). Decreased IR, decreased TIF (*P <* .0001), decreased EF (*P <* .01), and increased ML and EL (*P <* .05) were found in the model group when compared with the normal group. Tadalafil could reverse anhedonia through the sucrose preference test (*P <* .0001). IR (*P <* .001), TIF, and EF (*P <* .05) increased and EL decreased (*P <* .05) in the tadalafil group when compared with the model group; however, there was no difference in ML (*P >* .05). (Figure 2, Table 1).

**Table 1:** Sexual behavior parameters.

	Normal (n = 6)	Model (n = 7)	Tadalafil (n = 7)
ML, s	11.33 ± 1.93^a^	58.43 ± 13.38	63.71 ± 20.08
TIF	42.50 ± 5.48^b^	6.57 ± 1.74	22.00 ± 5.02^a^
TMF	8.83 ± 1.66	9.86 ± 1.68	6.14 ± 1.18
IR	0.83 ± 0.02^b^	0.38 ± 0.04	0.74 ± 0.02^b^
EL, s	1226 ± 215.60^a^	1800 ± 0.00	1269 ± 196.50^a^
EF	0.67 ± 0.21^a^	0	0.71 ± 0.29^a^

Values are mean ± SEM.

Abbreviations: EF, ejaculation frequency; EL, ejaculation latency; IR, intromission ratio; ML, mount latency; TIF, total intromission frequency; TMF, total mount frequency.

^a^ *P <* .05 vs the model group.

^b^ *P <* .0001 vs the model group.

### Effect of tadalafil on serum testosterone, dopamine, and 5-HT levels

**Figure 3:**
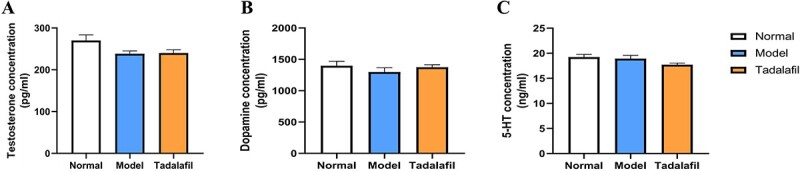
Serum testosterone, dopamine, and 5-HT measurement. (A) The level of serum testosterone (*F*_2,17_ = 3.414, *P >* .05). (B) The level of serum dopamine (*F*_2,17_ = 0.759, *P >* .05). (C) The level of serum 5-HT (*F*_2,17_ = 2.364, *P >* .05). Each value represents mean ± SEM.

Serum testosterone measured for different groups was as follows: normal group, 270.1 ± 13.52; model group, 238.5 ± 6.72; and tadalafil group, 240.2 ± 7.78. Serum dopamine measured for different groups was as follows: normal group, 1398 ± 70.18; model group, 1298 ± 67.31; and tadalafil group, 1373 ± 40.23. Serum 5-HT measured for different groups was as follows: normal group, 19.24 ± 0.55; model group, 18.92 ± 0.65; and tadalafil group, 17.71 ± 0.31. No significant differences were found in the levels of serum testosterone (*F*_(2,17_ = 3.414, *P >* .05), dopamine (*F*_(2,17_ = 0.759, *P >* .05), and 5-HT (*F*_2,17_ = 2.364, *P >* .05) among all groups (Figure 3).

## Discussion

In this study, rats exposed to CUMS displayed anhedonia and poor sexual function in terms of sucrose preference and sexual behavior. Meanwhile, we found that tadalafil could reverse anhedonia and prevent the changes of sexual function in rats induced by CUMS in accordance with the clinical treatment of tadalafil in pED, which offered a new perspective on the potential effects of tadalafil on the central nervous system. The method of CUMS is widely used to induce depression in rodents because of its high reliability and effectiveness. However, the protocol is complex and consumes more time, and ultimately is difficult to reproduce for other researchers. In a meta-analysis of model reliability about CUMS, the most frequent stressors listed included water and food deprivation, light cycle modification, soiled bedding, cage tilting, etc.^12^ Previous studies had demonstrated a strong association between depression and sexual function, indicating that major depressive disorder had high predictive value for sexual function.^22^^,^^23^ Chen et al^24^^,^^25^ reported that the white matter was damaged and the hub distribution of the left prefrontal and limbic cortex was abnormal in pED patients, while altered topological features of the functional brain networks were found by functional magnetic resonance imaging. Herein, a combination of different mild stressors was adopted, and the results confirmed our view that not only anhedonia, but also decreased sexual function occurred in CUMS-induced rats.

It is critical that female rats are included in the sexual behavior test for an objective assessment of male rats’ sexual function. Thus, ovariectomy, estrus induction, and a vaginal smear test were performed in female rats in this study. Results demonstrated that all female rats were not in estrus following ovariectomy. Estrus was successfully induced by progesterone and estradiol injection. Before CUMS, the apomorphine test was performed, and rats with no penile erection were removed. Rats with sexual experience were screened by sexual behavior tests conducted prior to the official test.

Significant differences were found in sexual behavior among 3 groups, suggesting that the sexual behavior test was a useful tool for evaluating sexual function. As an innate behavior,^26^ male mating behavior can be divided into 4 typical phases, including appetitive and consummatory phases. Male rats tentatively sniff the females’ external genitalia. The males mount the ladies by placing both forelimbs on their backs. If the females are agreeable, they will stay still and arch their backs. The intromission will proceed with the males’ hind legs and pelvis thrusting. Each intromission is brief and swift. Additionally, males are accustomed to licking their penises, which is regarded as a sign after intromission. Finally, it enters ejaculation at the same time as the final intromission, lasting the longest. The usual intromissions will increase in frequency before the final one. Along with the release of sperms, we may observe trembling in male rats.

The primary indicator of erectile function utilized in sexual behavior test is the IR parameter, which is typically used to screen for ED rats. The results of this study were consistent with another study that showed that IR typically ranged from 0.5 to 0.7 in normal rats.^27^ In parallel, there was a larger decrease in CUMS-induced rats, and IR could increase with the treatment of tadalafil, which suggested the reliability of IR in evaluating ED. Additionally, TIF showed significant variation among all groups. According to our findings, more intromissions occurred, indicating that male rats that engaged in copulation had stronger erectile function and more sexual desire. The current investigation confirmed that ML might be classified as sexual desire, in line with earlier findings.^28^ Actually, one of the key characteristics of pED was diminished libido, which manifested in extended ML during sexual behavior. As a result, the diminished erectile function and decreased sexual desire in CUMS-induced rats were consistent with the symptoms of pED patients. It was surprising to note that no ejaculation occurred in the CUMS-induced rats, which might be an important characteristic of pED rats. We assumed that the anhedonia and inhibition of ejaculation center induced by depression might explain why CUMS-induced rats were hard to attain the ejaculatory threshold.

Most research has concentrated on the effect of tadalafil on organic ED.^29^^,^^30^ Our results showed that tadalafil could prevent the changes induced by CUMS including anhedonia and poor sexual function.. In fact, tadalafil is a second-generation, highly selective phosphodiesterase type 5 inhibitor, which can enhance erectile function by increasing the content of cGMP in penile tissue. As a peripheral regulator, tadalafil has a variety of effects on different subtypes of ED.^31^ Despite pED having no obvious organic disease in the penis, its main mechanism is located in the central nervous system, which is still unclear.^32^ In fact, a normal erection is controlled by the central and peripheral nervous signal output,^33^ and the decreased, even dismissed nervous signal input and output will lead to ED. We assumed that tadalafil increased the peripheral function relatively to make up the deficiency of brain. In the clinic, tadalafil could also be used to treat pED in men. An observational study^34^ showed that daily tadalafil 5 mg could be helpful for ED patients with depressive symptoms and improved lower urinary tract symptoms and quality of life. An open-label, single-arm pilot study^35^ also suggested that daily low-dose tadalafil may have a potential role in the treatment of depression in patients with ED. Not only tadalafil, but also another phosphodiesterase type 5 inhibitor like sildenafil was reported to improve erectile function in men with sexual dysfunction associated with the use of serotonin reuptake inhibitor antidepressants.^36^ Interestingly, in accordance with the effect of tadalafil on antidepression, we found increased sucrose preference in the tadalafil group compared with the model group. A previous study^37^ reported that tadalafil could cross the blood-brain barrier, and is promising candidate for treatment of cognitive dysfunction. In fact, cognitive impairments were associated with induced depressive-like behaviors.^38^ Whether tadalafil could improve erectile function in CUMS-induced rats through ameliorating anhedonia remains unclear. Further behavior tests such as the open field test and forced swimming test are needed to measure the effect of tadalafil on antidepression.

There were several limitations in this study. According to relevant reports, testosterone and dopamine have a positive relationship with erectile function, while 5-HT has a strong link with depression.^39-41^ Although these biomarkers were examined in our study, we are disappointed that there were no differences in the levels of serum testosterone, dopamine, and 5-HT. We attempted to clarify the unfavorable outcomes. Following CUMS, serum levels of testosterone, dopamine, and 5-HT all fell, and tadalafil therapy showed a propensity to enhance all of these substances except 5-HT. In addition, for trustworthy and meaningful findings, it was important to further increase the number of rats in each group given the limited samples. Although CUMS-induced rats displayed anhedonia and poor sexual function in accordance with symptoms of pED, there are currently just a few studies on pED rats, which made it difficult to define the requirements for the pED model. Our research was an attempt, and future research needs to construct the standard of the pED model and explore the mechanism of tadalafil on central nervous system.

## Conclusion

It is highly advised that the method of CUMS and sexual behavior test should be used in the future for psychogenic sexual dysfunction modeling. In our investigation, a thorough knowledge of sexual behavior was demonstrated, and tadalafil could prevent the changes of depression and poor sexual function in rats induced by CUMS.

## Supplementary Material

supplementary_material_qfad019
