# Supplementary material for: Effects of tadalafil on sexual behavior of male rats induced by chronic unpredictable mild stress
Source: Sex Med. 2023 May 26;11(2):qfad019. doi: 10.1093/sexmed/qfad019 (PMC10225468; doi:10.1093/sexmed/qfad019)
Supplement: supplementary_material_qfad019 [file supplementary_material_qfad019.docx]

Supplementary Material

Effects of tadalafil on sexual behaviour of male rats induced by chronic unpredictable mild stress

Supplementary 1 Figure of sexual behaviour test and vaginal smear test.


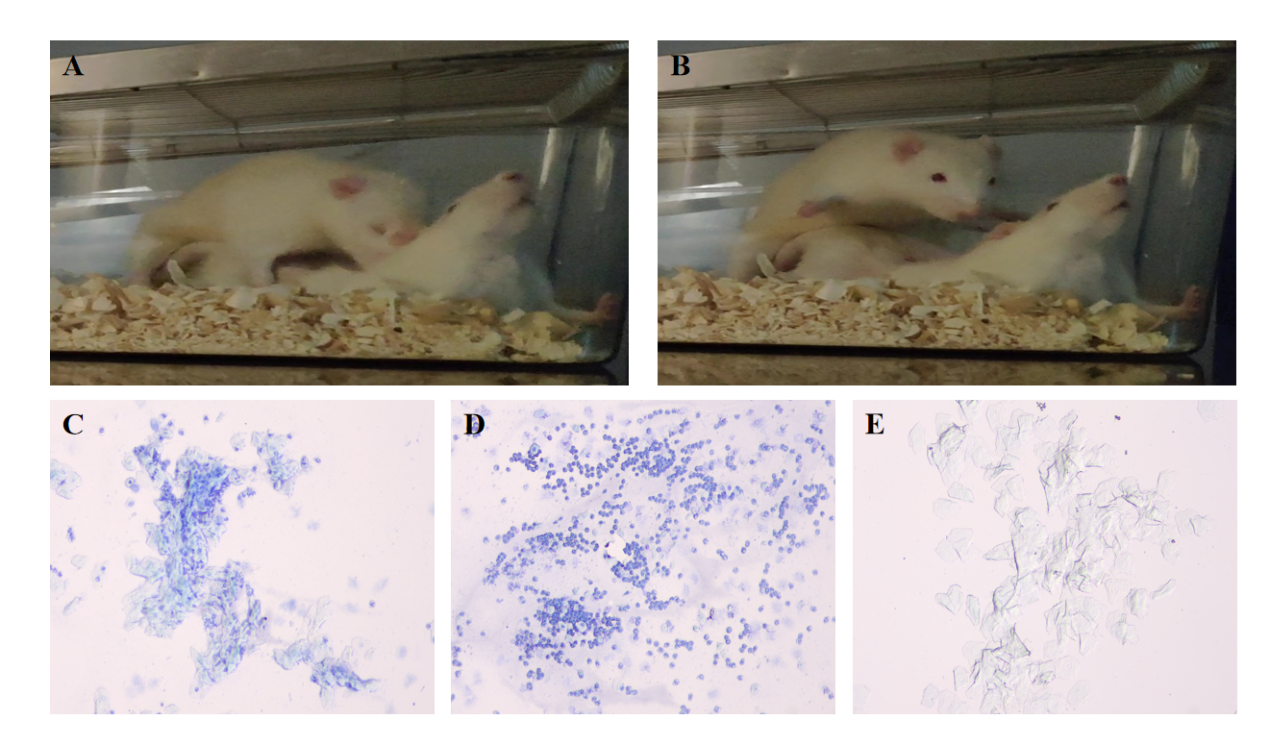


**Figure 1** Typical pictures captured in sexual behaviour test and vaginal smear test.(A-B) Two characteristic pictures during sexual behaviour test. (A) The deep and the last intromission before ejaculation. (B) The moment of ejaculation. (C-E) Vaginal smears of different estrous cycle. (C) A so-called epithelial “strand” containing high numebers of small nucleated epithelial cells, which is typical of proestrous. (D) Many neutrophils could be found in the diestrus. (E) A mass of anucleated epithelial cells respresented estrus. (C-D) After the ovariectomy. (E) After artificial induction of estrus. Toluidine blue stain was used. Original objective magnification of 10x.
